# Time series forecasting of COVID-19 infections and deaths in Alpha and Delta variants using LSTM networks

**DOI:** 10.1371/journal.pone.0282624

**Published:** 2023-10-20

**Authors:** Farnaz Sheikhi, Zahra Kowsari

**Affiliations:** Faculty of Computer Engineering, K. N. Toosi University of Technology, Tehran, Iran; VIT-AP Campus, INDIA

## Abstract

Since the beginning of the rapidly spreading COVID-19 pandemic, several mutations have occurred in the genetic sequence of the virus, resulting in emerging different variants of concern. These variants vary in transmissibility, severity of infections, and mortality rate. Designing models that are capable of predicting the future behavior of these variants in the societies can help decision makers and the healthcare system to design efficient health policies, and to be prepared with the sufficient medical devices and an adequate number of personnel to fight against this virus and the similar ones. Among variants of COVID-19, Alpha and Delta variants differ noticeably in the virus structures. In this paper, we study these variants in the geographical regions with different size, population densities, and social life styles. These regions include the country of Iran, the continent of Asia, and the whole world. We propose four deep learning models based on Long Short-Term Memory (LSTM), and examine their predictive power in forecasting the number of infections and deaths for the next three, next five, and next seven days in each variant. These models include Encoder Decoder LSTM (ED-LSTM), Bidirectional LSTM (Bi-LSTM), Convolutional LSTM (Conv-LSTM), and Gated Recurrent Unit (GRU). Performance of these models in predictions are evaluated using the root mean square error, mean absolute error, and mean absolute percentage error. Then, the Friedman test is applied to find the leading model for predictions in all conditions. The results show that ED-LSTM is generally the leading model for predicting the number of infections and deaths for both variants of Alpha and Delta, with the ability to forecast long time intervals ahead.

## 1 Introduction

Since December 2019 that an unknown devastating virus of the family of Coronavirus emerged, the whole world has been struggling to survive from the extreme circumstances. The new Coronavirus (2019-nCoV) can spread via airborne particles and droplets, and can cause severe lung involvement leading to death. On January 30, 2020, the World Health Organization (WHO) declared this outbreak, a Public Health Emergency of International Concern (PHEIC), and on February 11, 2020, WHO named this virus COVID-19. Soon after, on March 11, 2020, WHO declared it a pandemic [1]. The steep increase in the number of infections and deaths due to COVID-19 has made analysis on the transmission rate, algorithmic preventive solutions, and forecasting the statistics crucial to help decision makers to control the virus. Neither vaccines nor treatments were discovered at the early stage of COVID-19. Hence, preventive strategies such as travel bans, lockdowns, and quarantining suspected cases of COVID-19 received considerable attention [2, 3]. COVID-19 has mutated several times since its initiation, and different variants of the virus have arisen so far [4]. One of the most important differences in characteristics of COVID-19 variants is their transmissibility. The transmissibility of an infectious disease is usually measured by estimation of the *basic reproduction number* (*R*_0_). The basic reproduction number (*R*_0_) of an infectious disease is defined as the average number of secondary infections caused by a primary infection in a fully susceptible population [5, 6]. On January 23, 2020, WHO estimated *R*_0_ of COVID-19 to be in the range of 1.4–2.5. Following studies estimated *R*_0_ of COVID-19 from January 1 to February 7, 2020 to be in the range of 1.4–6.49 with a mean of 3.28 and a median of 2.79 [7]. Later studies focused on estimating *R*_0_ of variants of COVID-19.

Several variants of COVID-19 have been reported so far, where *Alpha* and *Delta* variants are the most notable ones to compare according to the transmissibility, severity of symptoms, and effectiveness of vaccines [8]. Alpha variant of COVID-19, which is also known as B.1.1.7 variant, was designated on December 8, 2020 in the United Kingdom [8]. The common symptoms of Alpha variant include cough, loss of smell and taste, fever, and muscle aches [9, 10]. The transmission rate of this variant has been increased by 43 to 90% compared to the initial COVID-19 generation [11]. The basic reproduction number (*R*_0_) of Alpha variant is measured during its activation period ranging from 2.2 to 6.1 in different countries and continents [11–14]. The efficiency of one dose of vaccines is reported 29.5% in the lowest and 88.1% in the highest case against Alpha variant [12, 15, 16]. Moreover, the efficiency of two doses of vaccines is in the range of 74.5% to 100% against this variant which approves the positive effect of vaccination [12, 15–17].

Delta variant, which is also known as B.1.617.2 variant, is another type of Variants of Concern (VOC), first found in October 2020 in India [8]. Cough and loss of smell are less reported in this variant, while cold-like symptoms including headache, sore throat, and a runny nose are more frequent in Delta variant in comparison to Alpha variant [9, 10]. Delta variant is more aggressive and transmissible than Alpha variant by 60%, with increased risk of hospitalization and death [18, 19]. The basic reproduction number (*R*_0_) of Delta variant is estimated in the range of 3.2–8 [13, 20]. The efficiency of one dose of vaccines against Delta variant is far from Alpha variant. It is shown that one dose of vaccines only provides 30.7% immunity against Delta variant [21]. However, having received the second dose of vaccines, the effectiveness of vaccines against Delta variant will increase to 88% [21]. Therefore, the findings support uptake of at least two vaccine doses to overcome Delta variant [21].

In Iran, COVID-19 was designated on February 19, 2020, when the Health Organization of Iran announced identification of two confirmed cases in the city of Qom [22]. Simultaneously, the Iranian government applied a vast variety of effective preventive strategies such as canceling public events [23], closing educational centers and shopping malls [24], plus setting traffic restrictions to control the spread of the virus. Similar to other countries, Iran also experienced different waves of COVID-19. Alpha variant of COVID-19 got active in Iran during the time interval from February 28 to June 9, 2021. Further, Delta variant was the dominant variant in Iran from June 10 to September 22, 2021. According to WHO website [8], in Asia and the Middle-East and worldwide, Alpha variant was active in the time interval from January 4 to May 15 and Delta variant was active from May 22 to September 22, 2021. Herein, forecasting COVID-19 statistics can help further understanding of nature of the virus and assist governments to tighten or loosen the restrictions. Moreover, it can be useful to plan for the required number of essential medical devises and drugs [25]. To this aim, several approaches like deep learning, linear models and other algorithms have been presented so far to forecast the number of infections and deaths of this ongoing pandemic.

Pinter et al. [26] proposed a Multi-Layered-Perceptron (MLP) integrated with an Imperialist Competitive Algorithm (ICA) for predicting the number of infections and mortality rate of COVID-19 in Hungary. To achieve a more accurate forecast, Zheng et al. [27] designed a hybrid AI model that is a combination of an Improved Susceptible-Infected (ISI) model, the Natural Language Processing (NLP) module, and the Long Short-Term Memory (LSTM) network. This model can predict COVID-19 statistics for the next six days in China.

For the prediction window of ten days in Pakistan, Khan et al. [28] proposed a Vector Autoregressive (VAR) model to predict the number of cases, recoveries, and deaths due to COVID-19 in the time interval from March 8 to June 27, 2020. Bayesian Dynamic Linear Model (BDLM) was also applied to forecast COVID-19 statistics in Pakistan for a longer prediction window of 20 days ahead from March 21 to April 9, 2021, based on the available data [29]. Khan et al. [29] showed in this study that the maximum number of cases, recoveries, and deaths due to COVID-19 in Pakistan during the aforementioned forecast interval would not exceed 4031, 3464, and 81, respectively. For assessing the progress of COVID-19 in Iran, Pakistan, and the neighboring countries, Feroze [30] proposed a Bayesian Structural Time Series (BSTS) model, and compared its predictive power with the Auto-Regressive Integrated Moving Average (ARIMA) models. The results approved superiority of BSTS model over classic ARIMA models.

The Adaptive Neuro-Fuzzy Inference System (ANFIS) and its extensions have shown strength in predicting various infectious diseases. Al-Qaness et al. [31] have provided an improved version of ANFIS by applying the Flower Pollination Algorithm (FPA) and the Sine Cosine Algorithm (SCA) in a model called FPASCA-ANFIS. The proposed FPASCA-ANFIS model was used to predict the weekly number of influenza cases in China and USA, which produced remarkable results. Performance of ANFIS was further examined for predicting the statistics of COVID-19 in different geographical regions. An improved version of ANFIS using a new nature-inspired optimizer, called Marine Predators Algorithm (MPA), was used to predict the number of infections in the countries of Italy, Iran, Korea, and USA [32]. ANFIS was further merged with the Salp Swarm Algorithm (SSA) and FPA to forecast the number of COVID-19 infections in China [33]. A combination of ANFIS with the chaotic MPA showed strength in COVID-19 predictions in Russia and Brazil [34].

Ayoobi et al. [35] forecasted the number of COVID-19 infections and deaths using six different deep learning methods. The methods include LSTM, Convolutional LSTM (Conv-LSTM), Gated Recurrent Units (GRU), and their bidirectional extensions. These methods were tested on COVID-19 datasets of Australia and Iran for predicting COVID-19 statistics for the next, the next three, and the next seven days. Dataset of Australia was studied in the time interval from January 25 to August 19, 2020 and dataset of Iran was considered in the time interval from January 3 to October 6, 2020. The results showed that in predicting the number of infections for the next day in Australia, LSTM and Bi-GRU have the best performance. However, for the next three, and next seven days, Conv-LSTM and Bi-Conv-LSTM outperform the other methods. In Iran, Bi-GRU showed better performance for predicting the number of confirmed cases for the next day, and the next three days while Bi-Conv-LSTM was better for predicting the next seven days. The results were slightly different for predicting the number of deaths. In predicting the number of deaths for the next day, Bi-GRU had the best performance in both countries of Iran and Australia. For the next three days, GRU was the superior model in predictions for Australia while Bi-Conv-LSTM was better for Iran. In predicting the next seven days, Bi-Conv-LSTM was still better for Iran while LSTM outperformed the other methods for the dataset of Australia.

Kafieh et al. [36] studied Random Forrest (RF), MLP, and various versions of LSTM such as LSTM with Regular Features (LSTM-R), LSTM with Extended Features (LSTM-E), and Multivariate LSTM (M-LSTM) to forecast COVID-19 statistics in the countries of China, Germany, Iran, Italy, Japan, Korea, Switzerland, Spain, and the USA. The time interval considered for evaluation was from January 22 until August 31, 2020. The results showed that M-LSTM outperformed the other models in the aforementioned countries.

Focusing on the factors such as age and facilities, ArunKumar et al. [37] proposed an RNN-LSTM and an RNN-GRU model to forecast the number of COVID-19 infections, recoveries, and deaths in the top ten countries based on the cumulative number of infections until August 2020. These countries were USA, Brazil, India, Russia, South Africa, Mexico, Peru, Chile, United Kingdom (UK), and Iran. The results showed that for predicting the number of confirmed cases and deaths, LSTM had achieved better results in the majority of these countries. For the recovered cases, GRU had better results for the majority of countries.

Kirbaş et al [38] provided a comparative analysis of effectiveness of ARIMA, Nonlinear Auto-Regression Neural Network (NARNN), and LSTM in forecasting the number of COVID-19 cases in eight European countries including Denmark, Belgium, Germany, France, United Kingdom, Finland, Switzerland, and Turkey. The time interval considered was from the first day of designation of COVID-19 in each of these countries until May 3, 2020. Comparison of the results showed that the dominant model was LSTM. LSTM also worked well for predicting the number of infections in Canada [39].

Verma, Mandal, and Gupta [40] examined the power of vanilla LSTM, stacked LSTM, Encoder Decoder LSTM (ED-LSTM), Bi-LSTM, Convolutional Neural Network (CNN), and hybrid CNN-LSTM models in forecasting the number of infections for the next 7, 14, and 21 days in India and its four most affected states. Among the aforementioned models, stacked LSTM and hybrid CNN-LSTM models showed a better performance in the majority of states on the dataset updated by July 10, 2021. Chandra, Jain, and Singh Chauhan [41] studied the power of LSTM, Bi-LSTM, and ED-LSTM in forecasting the number of COVID-19 infections for two months ahead (October and November 2021) in the top ten states of India (in terms of the number of infections). The results were slightly different for states with different population densities and cultures. However, ED-LSTM generally showed the best performance for predictions in states of India. Examining the power of this model in predicting COVID-19 statistics in other geographical regions was recommended for further research.

Although a deep body of research has been devoted to the study of COVID-19 and predicting its statistics since the designation of this virus, variant by variant, the virus has surprised researchers by proposing different behavior, structure, transmissibility, hospitalization, and mortality rate. Recent variants of COVID-19 have even shown immunity evasion in vaccinated people [42]. This empowers the assumption that although these variants have all inherited the main characteristics of the virus, each can have independent characteristics as well. Hence, in this paper, our goal is to examine the power of LSTM networks- namely, ED-LSTM, Bi-LSTM, Conv-LSTM and GRU- in predicting the number of infections and deaths in the two noticeably different variants of COVID-19: Alpha and Delta variant. Moreover, we have studied the effect of different geographical regions in terms of size, population, and cultural diversities on the predictive power of the models. We have considered geographical regions in the scale of a country (Iran), a continent (Asia), and the whole world for this analysis. To the best of our knowledge, this is the first time that these factors are put together to evaluate the performance of LSTM networks in the predictions. The results of this study can provide a broader vision on characteristics of the unknown nature of COVID-19 virus, and the possible future outbreaks related to the Coronavirus. This can help decision makers, medical manufacturing companies, and healthcare systems to be prepared with the sufficient quantity of human resources, medical devices, and infrastructures to react rapidly and efficiently against outbreaks of similar type.

The rest of the paper is organized as follows. In Section 2, datasets are introduced. Descriptions of details of the models ED-LSTM, Bi-LSTM, Conv-LSTM, and GRU are provided in Section 3. The evaluation metrics are explained in Section 4. Section 5 presents the results of the study. Section 6 provides a detailed analysis of the results and comparison of the models based on the geographical regions and variants. Finally, Section 7 concludes the paper, and presents recommendations for the future work.

## 2 Data

There are two datasets used in this paper to obtain the number of infections and deaths in the country of Iran, continent of Asia and the Middle East, and worldwide in the time intervals when Alpha and Delta variants of COVID-19 were the dominant variants. COVID-19 statistics of Iran for Alpha variant (Feb. 28—Jun. 9, 2021) and Delta variant (Jun. 10—Sept. 22, 2021) were obtained from the formal announcements of the Ministry of Health and Medical Education of Iran, published in the Iranian Students News Agency (ISNA) website [43]. COVID-19 statistics of the continent of Asia and the Middle East in Alpha and Delta variants were collected from the WHO website [44]. This site also provides worldwide cumulative number of cases and deaths in Alpha variant (Jan. 4—May 15, 2021) and Delta variant (May 22—Sept. 22, 2021). The beginning and the end of each time interval is determined based on the reports of WHO website [8]. According to the conducted studies [45], duration of the dominance of Delta variant has been longer than Alpha variant all over the world due to the different structure of this variant. Further, the active period of Alpha and Delta variants worldwide is longer than Iran due to differences in strictness of the preventive strategies such as remote working and social distancing in various countries and different vaccination rates. Hence, the time interval in which these variants were dominant in each of the target geographical regions is considered for the analysis.

One of the challenges to work with COVID-19 datasets is presence of remarkable noise in the daily statistics. This inaccuracy in the reported statistics is dependent to several factors such as unavailability of the sufficient number of diagnostic kits for a specific day, inherent error of the kits, and the failure to correctly record the date of illness due to holidays or late visit to the healthcare system. Hence, a currently infected person may be enumerated in the statistics of few days later. Thus, working with daily COVID-19 statistics is not quite reliable. For this reason, we have used the cumulative number of infections and deaths in this paper to lessen the effect of inaccuracy of the data in comparison of the predictive power of the models. The datasets are divided into a training set (75% of data) on which our models are trained, and a test set (25% of data). Further, 20% of the training set is used for validation. After choosing the best model in each of the geographical regions, to show generality of the models, we have forecasted the emergence of a new wave after Alpha and Delta variants; See Section 6. To this aim, we have worked on the daily statistics of COVID-19. In this case, to ignore the sudden fluctuations in the daily statistics, we have smoothed out these datasets by using the centered moving average method.

## 3 Methods

### 3.1 LSTM and Encoder Decoder LSTM

**Fig 1:**
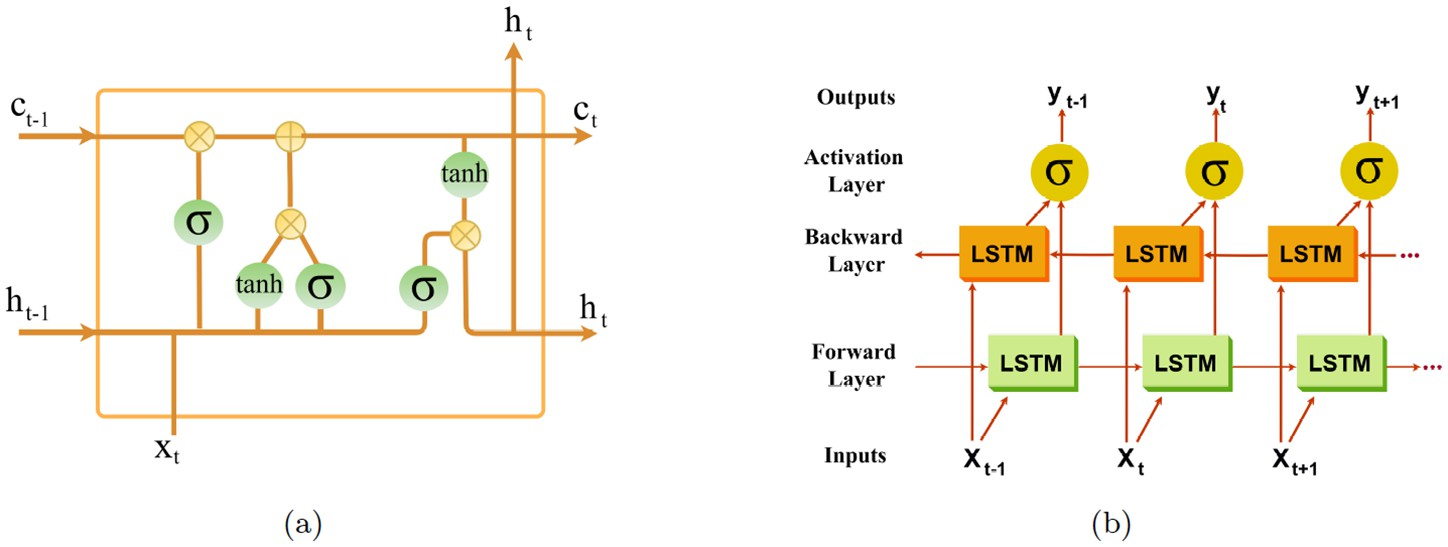
(a) An LSTM cell (b) Architecture of a Bi-LSTM.

The famous Recurrent Neural Network (RNN) is a type of artificial neural network that allows use of previous outputs as input of the current step by providing back-propagation. Despite strength of RNNs, the problem of vanishing gradient prevents these networks from learning long-term dependencies. The most effective solution to tackle this issue is to use memory cells in Long-Short Term Memory (LSTM) networks. LSTM is a type of RNN that is able to add or remove information to the cell state via three multiplicative gates: *input gate, output gate*, and *forget gate*. For the current input *x*_*t*_ and historical information *h*_*t*−1_ coming from the previous step, forget gate *f*_*t*_ decides which information should be thrown away (forgotten) from the cell state. This decision is made by a Sigmoid layer, denoted by *σ*. On the other hand, the input gate *i*_*t*_ decides which information should be stored (remembered) in the cell state. The Sigmoid layer decides the values to be updated while tanh layer weighs these values to be added to the state ${\tilde{c}}_{t}$. Now, it is time to update the previous cell state *c*_*t*−1_ to the new cell state *c*_*t*_ by combining the values generated in the forget gate and input gate. The output gate *o*_*t*_, which is the last gate in the architecture, decides which should be given as output. This gate also has a Sigmoid layer for selection and a tanh layer to produce the final output. See Fig 1(a). The corresponding equations are as follows.

$$\begin{array}{cc} f_{t} & =\sigma (w_{f}\cdot \left[h_{t-1},x_{t}\right]+b_{f})\\ i_{t} & =\sigma (w_{i}\cdot \left[h_{t-1},x_{t}\right]+b_{i})\\ {\tilde{c}}_{t} & =\text{tanh}(w_{c}\cdot \left[h_{t-1},x_{t}\right]+b_{c})\\ c_{t} & =f_{t}\cdot c_{t-1}+i_{t}\cdot {\tilde{c}}_{t}\\ o_{t} & =\sigma (w_{o}\cdot \left[h_{t-1},x_{t}\right]+b_{o})\\ h_{t} & =o_{t}\cdot \text{tanh}\left(c_{t}\right) \end{array}$$

Encoder Decoder LSTM (ED-LSTM) is the combination of an encoder and a decoder where both the encoder and decoder are LSTMs. Herein, an encoder LSTM reads the input and summarizes it into state vectors. These state vectors are then fed into a decoder LSTM to generate the output. This model is used in multi-step time series predictions when the length of input and the length of output are different [46].

### 3.2 Bidirectional LSTM

Bidirectional LSTM (Bi-LSTM) is an extension of the traditional LSTM with the capability of running inputs in both forward and backward directions. Namely, in a Bi-LSTM, apart from a regular LSTM, there exists a complementary LSTM layer to process the information backwards. This bidirectional processing increases the amount of information available to the network. Fig 1(b) illustrates architecture of a Bi-LSTM.

### 3.3 Convolutional LSTM

Convolutional LSTM (Conv-LSTM) is a combination of Convolutional Neural Networks (CNNs) and LSTMs. In this architecture, CNN layers are used for feature extraction, and LSTM layers are used for sequence prediction. The mathematics behind a Conv-LSTM are very similar to an LSTM except addition of the convolution operator *:

$$\begin{array}{cc} f_{t} & =\sigma (w_{f}\cdot \left[h_{t-1},x_{t}\right]+b_{f})\\ i_{t} & =\sigma (w_{i}\cdot \left[h_{t-1},x_{t}\right]+b_{i})\\ g_{t} & =\text{tanh}(w_{c}\cdot \left[h_{t-1},x_{t}\right]+b_{c})\\ c_{t} & =f_{t}\cdot c_{t-1}+i_{t}\cdot g_{t}\\ o_{t} & =\sigma (w_{o}\cdot \left[h_{t-1},x_{t}\right]+b_{o})\\ h_{t} & =o_{t}*\text{tanh}\left(c_{t}\right) \end{array}$$

### 3.4 Gated Recurrent Unit

**Fig 2:**
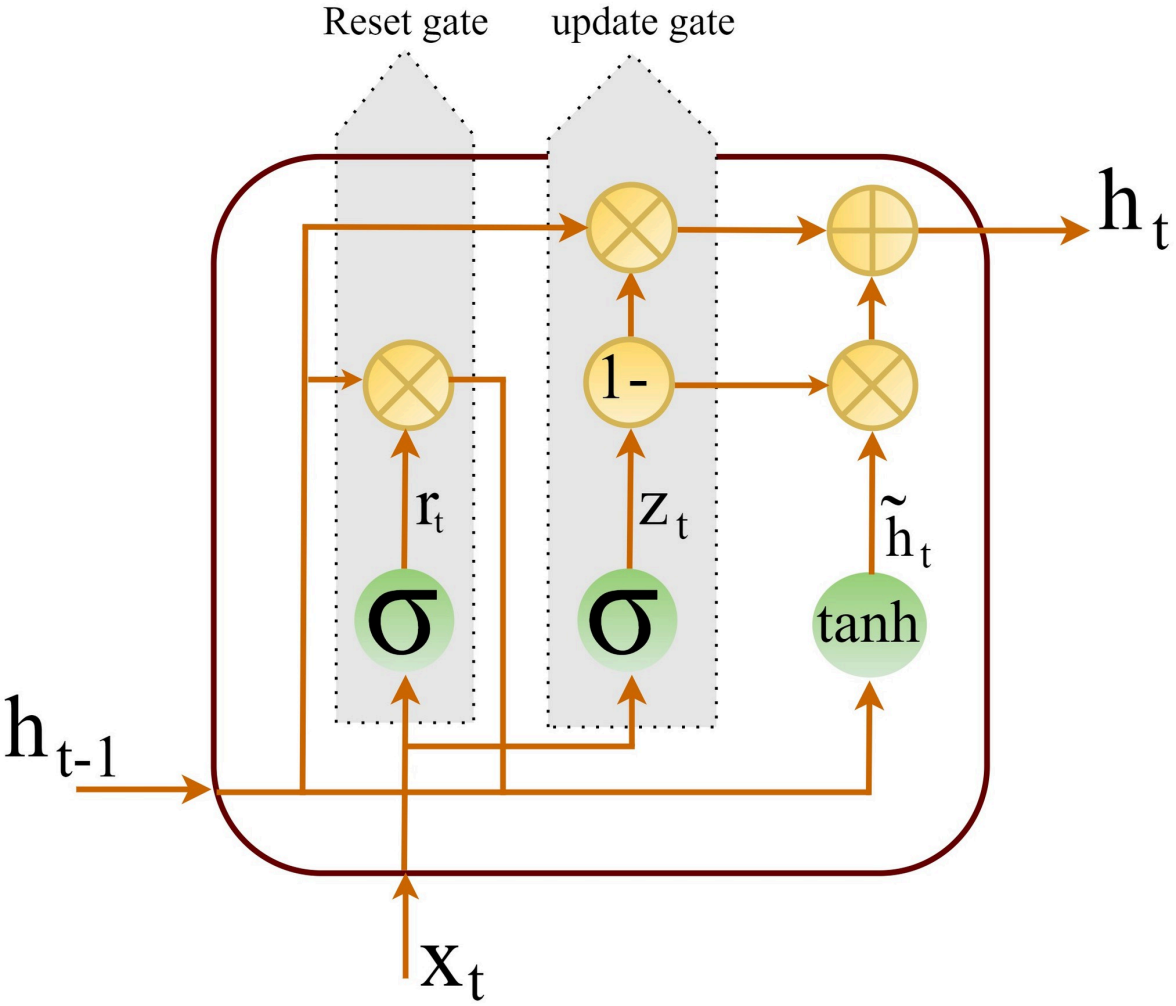
General structure of a GRU.

Gated Recurrent Unit (GRU) is an improvement over LSTM with fewer parameters and operations. To solve the vanishing gradient problem without the overhead of standard LSTMs, GRU uses only two gates: *update gate* and *reset gate*. The update gate decides which amount of the past information should be passed to the future. This way, any feature which is recognized important, can be retained without being overwritten or lost. Then, this gate creates shortcuts to skip through several time steps and overcome the vanishing gradient problem. Indeed, the update gate in a GRU is a combination of the input gate and the forget gate in an LSTM. The other gate, namely the reset gate in GRU, is used to decide how much of the past information should be forgotten. Fig 2 illustrates architecture of a GRU. The mathematics behind GRU come next.

$$\begin{array}{cc} r_{t} & =\sigma (W_{r}\cdot x_{t}+U_{r}\cdot h_{t-1}+b_{r})\\ z_{t} & =\sigma (W_{z}\cdot x_{t}+U_{z}\cdot h_{t-1}+b_{z})\\ \tilde{h_{t}} & =\phi_{h}\left(W_{h}\cdot x_{t}+U_{h}\cdot \left(r_{t}⊙h_{t-1}\right)+b_{h}\right)\\ h_{t} & =\left(1-z_{t}\right)⊙h_{t-1}+z_{t}⊙\tilde{h_{t}} \end{array}$$

Where *x*_*t*_ and *h*_*t*_ are the input and the output vector, respectively. Candidate activation vector is denoted by $\tilde{h_{t}}$. The operator ⊙ represents the Hadamard product. The update gate vector and the reset gate vector is respectively denoted by *z*_*t*_ and *r*_*t*_. Finally, *W*, *U*, and *b* represent parameter matrices and a vector.

## 4 Evaluation metrics

To compare the performance of the aforementioned networks, we have used the following three evaluation metrics: Root Mean Square Error (RMSE), Mean Absolute Error (MAE), and Mean Absolute Percentage Error (MAPE).

RMSE is one of the popular evaluation metrics to assess the quality of forecasting models. It measures the standard deviation of the prediction errors, and can be used as an important criterion to specify the best forecasting model. Eq 1 provides a detailed definition of this evaluation metric. Squaring the prediction errors, RMSE highlights large errors. However, in MAE, prediction errors are not weighted. The MAE measures the average of the absolute prediction errors, as described in Eq 2. MAPE measures the percentage of relative errors, defined in Eq 3. This way, a unitless measure will be obtained that is suitable for reporting the results. Considering these three metrics provides a comprehensive comparison of the forecasting models.

$$\begin{array}{c} RMSE=\sqrt{\frac{\sum_{i=1}^{n}{\left(y_{t}-{\hat{y}}_{t}\right)}^{2}}{n}} \end{array}$$ (1)

$$\begin{array}{c} MAE=\frac{\sum_{i=1}^{n}\left(y_{t}-{\hat{y}}_{t}\right)}{n} \end{array}$$ (2)

$$\begin{array}{c} MAPE=\frac{100*\sum_{i=1}^{n}\left(\frac{y_{t}-{\hat{y}}_{t}}{y_{t}}\right)}{n} \end{array}$$ (3)

In Eqs 1, 2 and 3, *n* is the size of the dataset under study, *y*_*t*_ denotes the actual value, and ${\hat{y}}_{t}$ denotes the predicted value.

## 5 Results

The proposed models are implemented in Python programming language using Jupiter Notebook app, run on a Lenovo Z400 touch, with 8GB RAM, 1TB SSD, Intel Core i7-3632QM 2.20GHz Quad-Core CPU, and Windows 10. Predictions of the models have been repeated 100 times, and the one with the least prediction error is taken.

ED-LSTM, Bi-LSTM, Conv-LSTM, and GRU models are used to predict the number of infections and deaths in Alpha and Delta variants of COVID-19 for several time steps ahead. Performance of these models in predictions are also evaluated in different geographical regions: country of Iran, continent of Asia and the Middle East, and the whole world. The architecture of our ED-LSTM includes two-encoder and two-decoder layers with 100 LSTM units in each layer. Adam optimizer is used with Huber loss function. Moreover, since the output size of the encoder and the input size of the decoder are different, a RepeatVector layer is added for the connection. Our Bi-LSTM has an input layer with 150 LSTM units plus two hidden layers with 128 LSTM units. The architecture of our Conv-LSTM includes a single layer Conv-LSTM with 64 filters. The architecture of our GRU includes an input layer with 64 neurons and a hidden layer with the same number of neurons. Adam optimizer is used in the last three models with the mean squared error as the loss function.

In our ED-LSTM, Bi-LSTM, and GRU, we have considered the sequence size to be five. Hence, these models take every five days as the input and forecast the next three, the next five, and the next seven days. The forecasting time windows are the same for Conv-LSTM, with the difference that the sequence size in Conv-LSTM is four. It should be noted that the number of neurons in the output layer of each network varies between three, five, and seven, according to the aim of prediction. Further, 75% of the data is used to train, and 25% of data is used for test. Moreover, 20% of the training data is used for validation. The number of epochs to be considered is 100.

**Fig 3:**
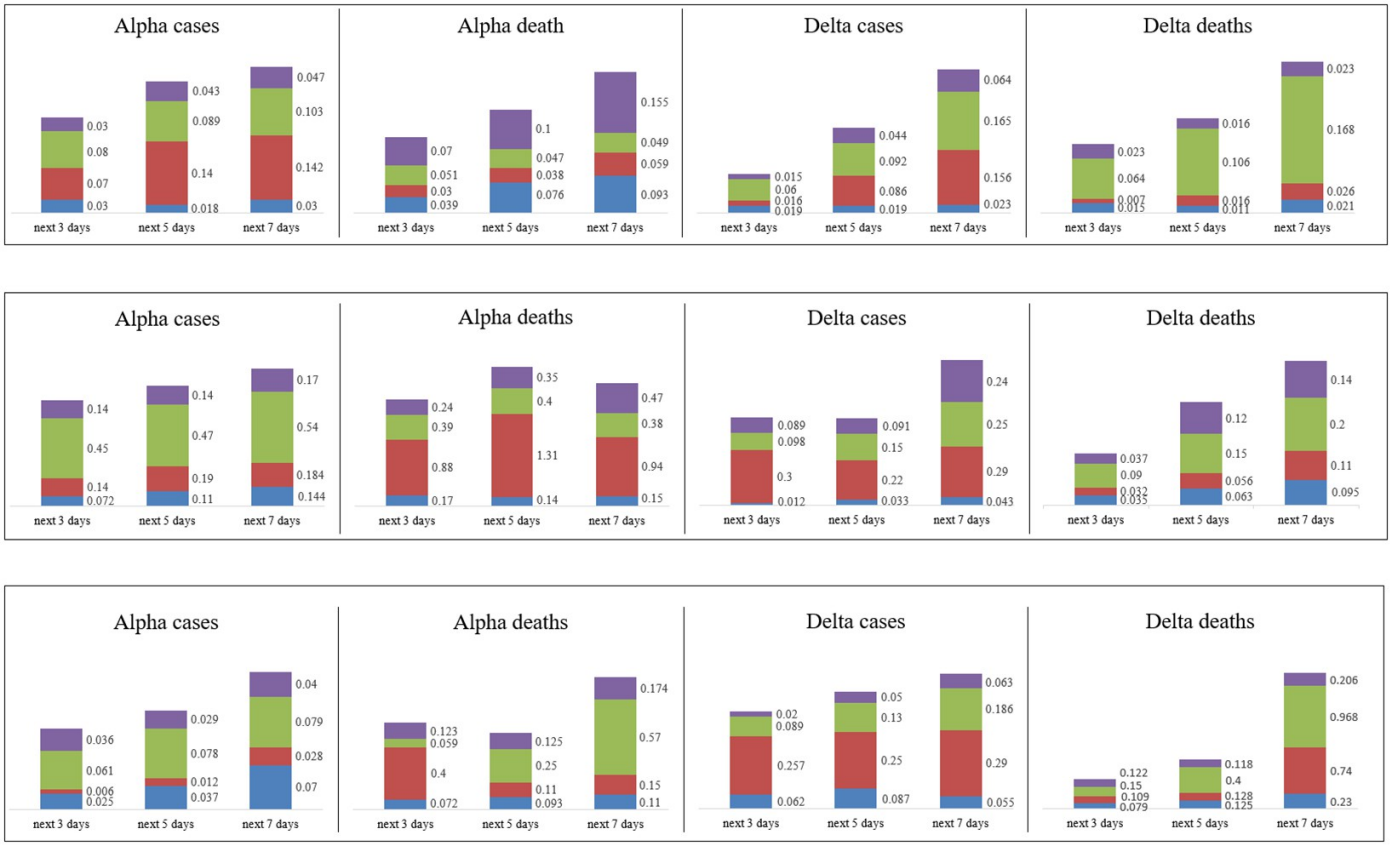
Graphical diagram of MAE corresponding to predicting the number of infections and deaths for the next three, the next five, and the next seven days of Alpha and Delta variants of COVID-19. Top down: the first row is corresponding to the MAE for predictions in the whole world, the second row is corresponding to the MAE for predictions in the continent of Asia and the Middle East, and the third row is corresponding to the MAE for predictions in the country of Iran. MAE of ED-LSTM, Bi-LSTM, Conv-LSTM, and GRU is respectively shaded blue, red, green, and purple.

**Fig 4:**
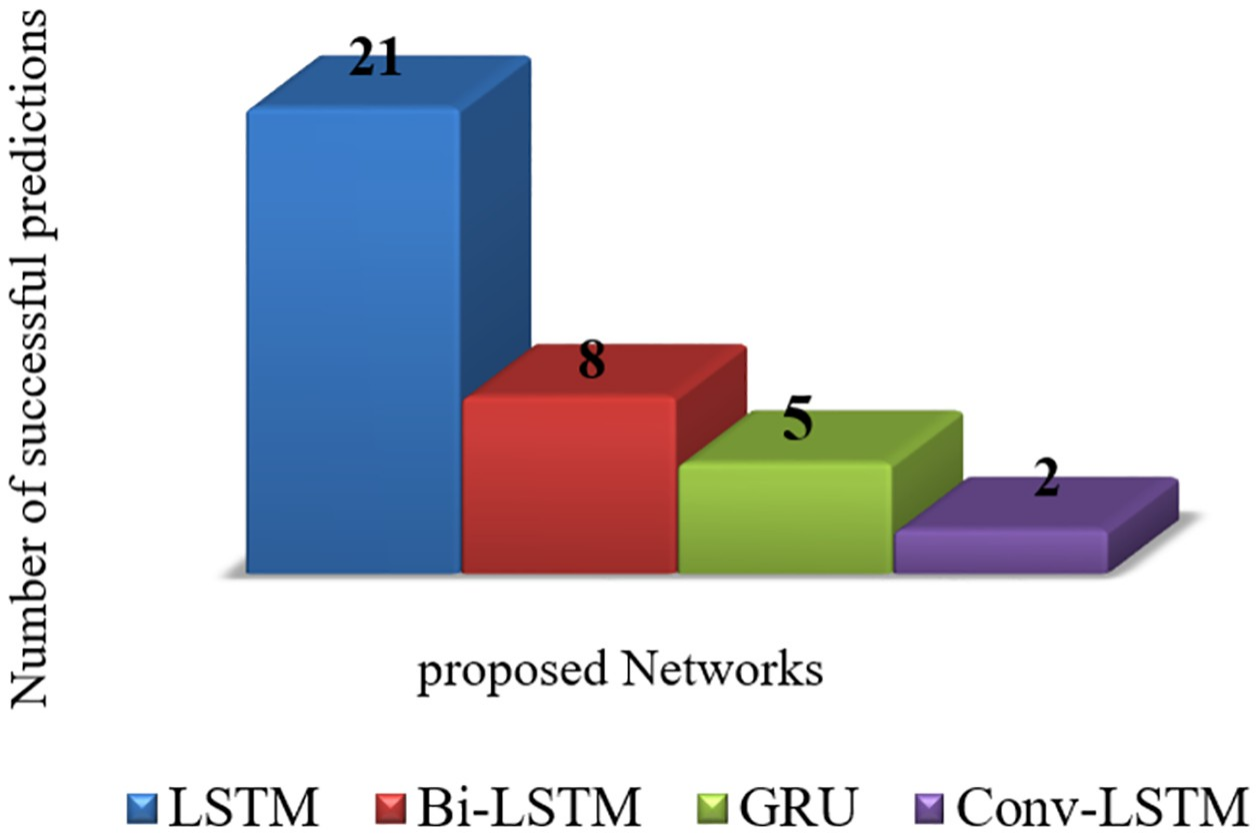
Share of each network in predictions with the least error.

To provide a comprehensive comparison of performance of the proposed models in predicting the number of infections and deaths, RMSE and MAPE are calculated and reported in Tables 1–3. Fig 3 illustrates the graphical diagram of MAE for the proposed models. Moreover, Fig 4 depicts the share of each of the proposed networks in predictions with the least error.

**Table 1:** RMSE and MAPE of predicting the number of infections and deaths for the next three, the next five, and the next seven days of Alpha and Delta variants of COVID-19 worldwide.

	Variants	Models	Next 3 days		Next 5 days		Next 7 days	
			RMSE	MAPE	RMSE	MAPE	RMSE	MAPE
**The World**	Alpha cases	ED-LSTM	**0.013**	**0.722**	**0.0195**	**1.059**	**0.033**	**1.77**
		Bi-LSTM	0.085	4.50	0.154	8.45	0.154	8.38
		Conv-LSTM	0.099	4.89	0.113	5.24	0.13	6.16
		GRU	0.039	1.99	0.048	2.6	0.053	2.83
	Alpha deaths	ED-LSTM	0.047	2.45	0.083	4.95	0.1	6.099
		Bi-LSTM	**0.036**	**1.96**	**0.044**	**2.46**	0.065	3.90
		Conv-LSTM	0.064	3.44	0.058	3.11	**0.059**	**3.38**
		GRU	0.074	4.67	0.108	7.23	0.159	10.53
	Delta cases	ED-LSTM	0.024	1.17	**0.023**	**1.23**	**0.025**	**1.593**
		Bi-LSTM	**0.017**	1.04	0.089	5.63	0.16	10.29
		Conv-LSTM	0.071	3.99	0.113	5.87	0.187	10.53
		GRU	**0.017**	**0.98**	0.45	2.86	0.065	4.15
	Delta deaths	ED-LSTM	0.017	1.02	**0.013**	**0.74**	**0.022**	**1.5**
		Bi-LSTM	**0.008**	**0.47**	0.016	1.14	0.027	1.87
		Conv-LSTM	0.08	4.26	0.124	7.09	0.19	11.35
		GRU	0.025	1.63	0.018	1.14	0.024	1.6

**Table 2:** RMSE and MAPE of predicting the number of infections and deaths for the next three, the next five, and the next seven days of Alpha and Delta variants of COVID-19 in Asia and the Middle East.

	Variants	Models	Next 3 days		Next 5 days		Next 7 days	
			RMSE	MAPE	RMSE	MAPE	RMSE	MAPE
**Asia and the Middle East**	Alpha cases	ED-LSTM	**0.088**	**2.54**	**0.13**	**4.07**	**0.162**	**5.37**
		Bi-LSTM	0.176	4.850	0.25	6.358	0.23	6.39
		Conv-LSTM	0.53	18.8	0.55	19.93	0.57	23.43
		GRU	0.169	4.67	0.19	4.74	0.20	6.05
	Alpha deaths	ED-LSTM	**0.2**	**6.75**	**0.17**	**4.8**	**0.16**	**5.56**
		Bi-LSTM	1.03	32.54	0.78	24.4	1.09	34.56
		Conv-LSTM	0.54	15.29	0.521	15.84	0.48	15.5
		GRU	0.26	9.1	0.37	13.91	0.5	18.57
	Delta cases	ED-LSTM	**0.013**	**0.81**	**0.038**	**2.13**	**0.048**	**2.77**
		Bi-LSTM	0.032	2.03	0.23	15.07	0.3	19.78
		Conv-LSTM	0.12	6.36	0.17	9.52	0.273	16.4
		GRU	0.091	5.96	0.094	6.1	0.24	16.28
	Delta deaths	ED-LSTM	0.035	2.46	0.064	4.5	**0.096**	**6.73**
		Bi-LSTM	**0.034**	**2.23**	**0.057**	**3.88**	0.12	7.93
		Conv-LSTM	0.1	6.15	0.166	10.37	0.22	14.36
		GRU	0.038	2.57	0.085	5.71	0.14	9.68

**Table 3:** RMSE and MAPE of predicting the number of infections and deaths for the next three, the next five, and the next seven days of Alpha and Delta variants of COVID-19 in Iran.

	Variants	Models	Next 3 days		Next 5 days		Next 7 days	
			RMSE	MAPE	RMSE	MAPE	RMSE	MAPE
**Iran**	Alpha cases	ED-LSTM	0.029	1.93	0.4	2.88	0.072	5.48
		Bi-LSTM	**0.006**	**0.44**	**0.014**	**0.94**	**0.029**	**2.1**
		Conv-LSTM	0.069	4.59	0.085	5.87	0.08	6.1
		GRU	0.041	2.7	0.033	2.17	0.045	3
	Alpha deaths	ED-LSTM	0.074	5.1	**0.097**	**6.57**	**0.11**	**7.77**
		Bi-LSTM	0.4	2.86	0.11	8	0.155	10.8
		Conv-LSTM	**0.07**	**4.11**	0.25	18.2	0.57	40.8
		GRU	0.124	8.7	0.127	8.67	0.176	12
	Delta cases	ED-LSTM	0.066	4.09	0.9	5.73	**0.059**	**3.6**
		Bi-LSTM	0.263	17	0.258	16.5	0.296	19.06
		Conv-LSTM	0.11	5.89	0.143	8.9	12.96	0.191
		GRU	**0.021**	**1.36**	**0.051**	**3.37**	0.064	4.16
	Delta deaths	ED-LSTM	**0.088**	**4.17**	0.137	6.75	0.24	12.36
		Bi-LSTM	0.12	5.81	0.138	7.14	0.743	42.38
		Conv-LSTM	0.18	8.53	0.401	23.03	0.976	55.22
		GRU	0.127	6.78	**0.12**	**6.6**	**0.23**	**11.33**

**Fig 5:**
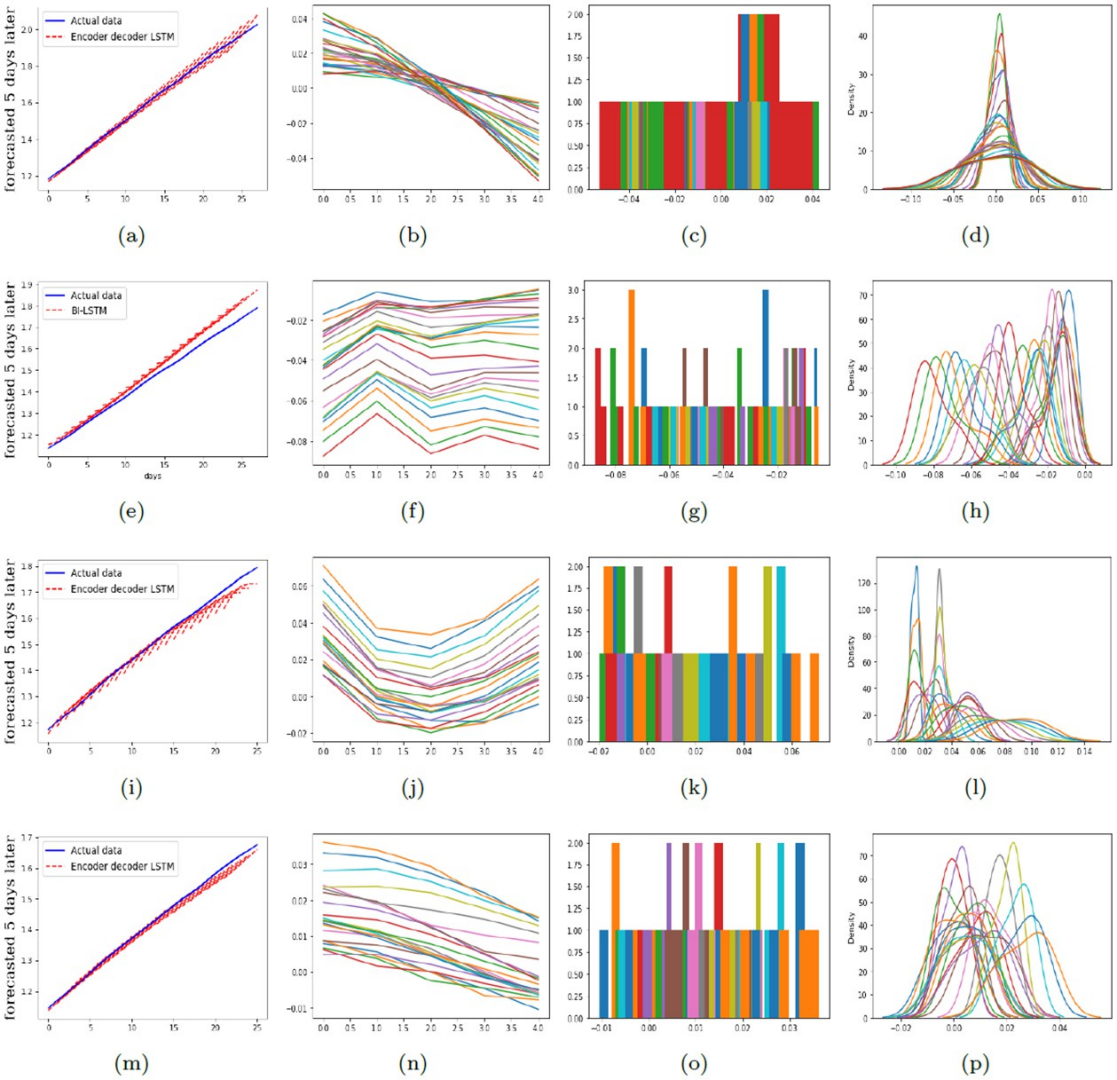
The residual, histogram, and density plots corresponding to the best model for predicting the number of infections and deaths in Alpha and Delta variants of COVID-19 in the whole world. Top down: the first row is corresponding to the number of infections in Alpha variant, the second row is corresponding to the number of deaths in Alpha variant, the third row is corresponding to the number of infections in Delta variant, and the fourth row is corresponding to the number of deaths in Delta variant. Further, from left to right: the second column depicts the residual plots, the third column illustrates the histograms, and the fourth column depicts the density plots.

**Fig 6:**
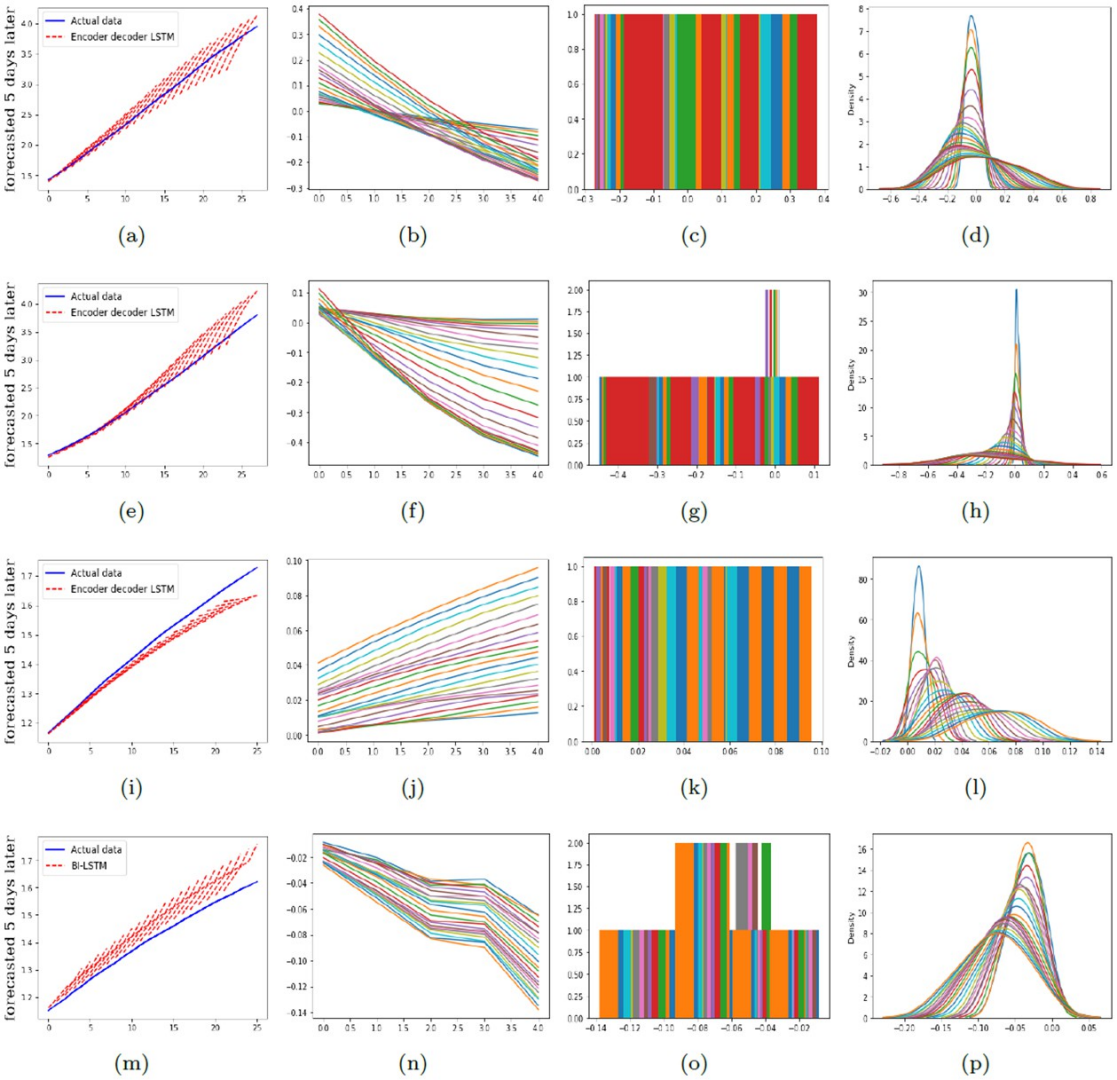
The residual, histogram, and density plots corresponding to the best model for predicting the number of infections and deaths in Alpha and Delta variants of COVID-19 in Asia. Top down: the first row is corresponding to the number of infections in Alpha variant, the second row is corresponding to the number of deaths in Alpha variant, the third row is corresponding to the number of infections in Delta variant, and the fourth row is corresponding to the number of deaths in Delta variant. Further, from left to right: the second column depicts the residual plots, the third column illustrates the histograms, and the fourth column depicts the density plots.

**Fig 7:**
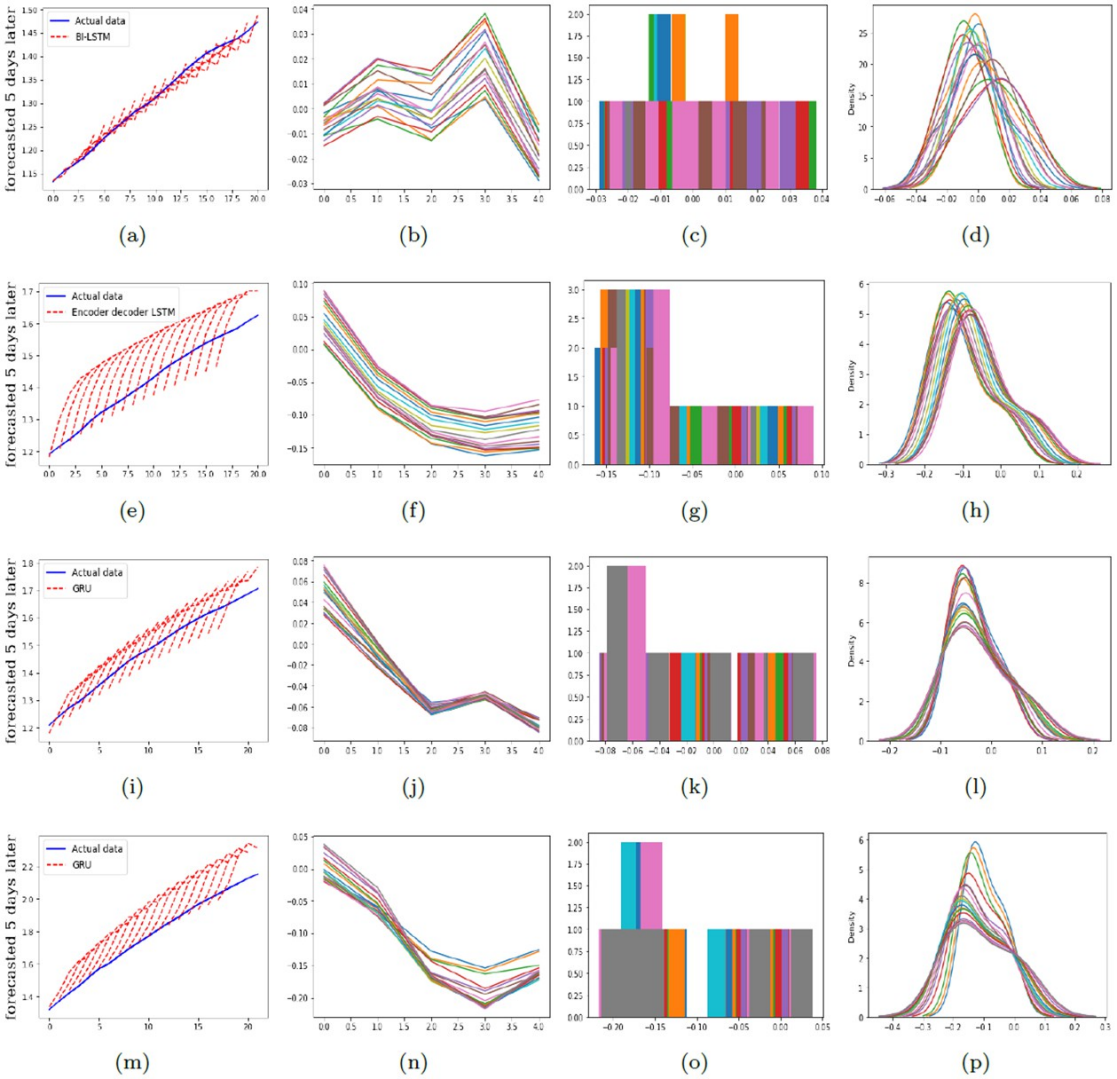
The residual, histogram, and density plots corresponding to the best model for predicting the number of infections and deaths in Alpha and Delta variants of COVID-19 in Iran. Top down: the first row is corresponding to the number of infections in Alpha variant, the second row is corresponding to the number of deaths in Alpha variant, the third row is corresponding to the number of infections in Delta variant, and the fourth row is corresponding to the number of deaths in Delta variant. Further, from left to right: the second column depicts the residual plots, the third column illustrates the histograms, and the fourth column depicts the density plots.

In Figs 5–7, the residual plots corresponding to the predictions with the least error are shown. The residual plot shows the difference between the observed value and the predicted value. In most cases, these deviations are minor and acceptable. However, the longer the forecast period becomes, the more this deviation increases. The histograms and the density diagrams in Figs 5–7 show that the residuals related to predicting the number of infections and deaths are approximately normally distributed.

## 6 Discussion

### 6.1 Examining the effect of diversity and size of a geographical region in predictions

#### Iran

As recorded in Table 3, among the proposed models, Bi-LSTM has achieved the best predictions for the number of infections in Alpha variant of COVID-19 in Iran. In predicting the number of deaths due to this variant, ED-LSTM has outperformed the other models. Considering Delta variant of COVID-19 in Iran, GRU model has outperformed the other models in predicting the number of infections and deaths. ED-LSTM has ranked second in predicting the number of infections and deaths due to Delta variant of COVID-19 in Iran.

#### Asia and the Middle East

Considering a larger geographical and more populated area with great diversity in economical and cultural aspects, namely the continent of Asia, ED-LSTM has outperformed the rest of models in predicting the number of infections and deaths in both variants of Alpha and Delta, as shown in Table 2. Bi-LSTM has achieved the second rank in predictions in this geographical region.

#### World

Considering the largest geographical area that is the whole world, ED-LSTM has shown great performance in predicting the number of infections and deaths in both variants of Alpha and Delta. See Table 1.

As the results in Tables 1 and 2 indicate, considering large geographical regions with great diversity in infrastructures, economical, and cultural aspects, such as the whole world and the continent of Asia, ED-LSTM networks show great performance in capturing the patterns and predicting the number of infections and deaths in both Alpha and Delta variants of COVID-19. Bi-LSTM achieves the second rank in these predictions, and GRU gets the third rank. The smaller the geographical region under the study gets, the more powerful Bi-LSTM and GRU networks become in predicting the number of infections and deaths. Hence, as shown in Table 3, for the geographical region of the country of Iran, Bi-LSTM and GRU respectively achieves the first and the second rank in predictions. Increasing the prediction time-window from three to seven days, it is natural that the error of prediction increases.

### 6.2 Examining the effect of structural difference in variants in predictions

#### Alpha variant

As recorded in Tables 1–3, ED-LSTM and Bi-LSTM has respectively achieved the first and the second rank in predicting the number of infections and deaths due to Alpha variant of COVID-19.

#### Delta variant

When considering Delta variant of COVID-19, ED-LSTM, Bi-LSTM, and GRU models achieve a slightly different performance in predictions. While ED-LSTM still takes the first rank in predictions, GRU herein gets the second rank in predictions, and Bi-LSTM is ranked third. See Tables 1–3.

As the results indicate, structural differences in COVID-19 variants can also affect the power of predictions in LSTM networks. Putting these factors all together, next we propose a statistical analysis to specify the best LSTM network for predictions in all conditions, and continue forecasting the statistics of COVID-19 with the winer model.

### 6.3 Statistical analysis

So far, we have examined the performance of ED-LSTM, Bi-LSTM, Conv-LSTM, and GRU models in predicting the number of infections and deaths due to Alpha and Delta variants of COVID-19, using RMSE, MAPE and MAE evaluation metrics. To provide a general comparison of these models to choose the best model, herein we use the non-parametric statistical test of Friedman test [47]. For this test, the average value of the evaluation metrics are calculated, and then the proposed models are ranked based on this average, using the Friedman test. The results of this ranking are recorded in Table 4.

**Table 4:** The ranking of LSTM networks in predicting the number of infections and deaths.

	First Rank	Second Rank	Third Rank	Fourth Rank
Alpha variant	ED-LSTM	Bi-LSTM, GRU	Conv-LSTM	-
Delta variant	ED-LSTM	GRU	Bi-LSTM	Conv-LSTM
Iran	GRU	ED-LSTM	Bi-LSTM	Conv-LSTM
Asia and the Middle East	ED-LSTM	GRU	Bi-LSTM	Conv-LSTM
Global	ED-LSTM	Bi-LSTM	GRU	Conv-LSTM

As concluded from the results of the Friedman test in Table 4, ED-LSTM has achieved the first rank for predictions in all conditions, both according to the type of variant and according to the geographical location. We continue forecasting the number of infections and deaths for the next two months with the winning ED-LSTM network.

### 6.4 Forecasting the future

Using the models that are capable of forecasting the future waves of an infectious disease, we will be prepared to increase the level of safety of people and the society by announcing the necessary warnings and preventive strategies to the people. Policy makers and healthcare managers can further benefit from these statistics by providing necessary facilities and strategies to reduce the peak of the future waves significantly. Due to importance of this issue, in this paper, we have further examined the generality and reliability of ED-LSTM network in forecasting the next wave in two months, following each of Alpha and Delta variants of COVID-19.

**Fig 8:**
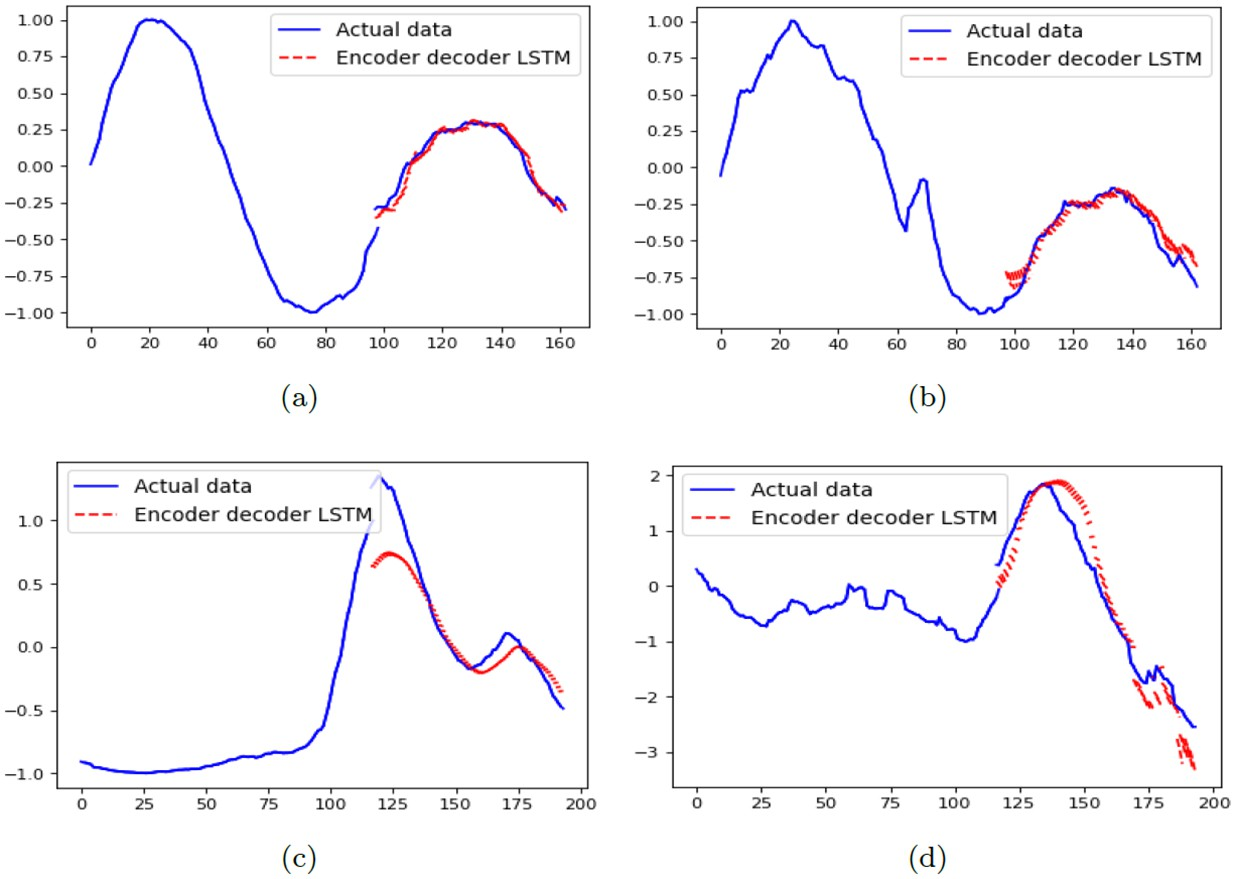
Predicting the number of infections and deaths due to COVID-19 for the next two months following Alpha and Delta variants in the world. Top down: the first raw is corresponding to the statistics of Alpha variant, and the second raw is corresponding to the statistics of Delta variant. From left to right: the first column is corresponding to the number of infections, and the second column is corresponding to the number of deaths.

The results of forecast are depicted in Fig 8, which clearly show that ED-LSTM networks have the ability to forecast the future waves. There are also cases that the forecasted pick is lower than the actual pick. This resembles the situation in the society that leaving behind a deadly variant, with the decrease in the number of infections and deaths, people neglect following strict health protocols. This general negligence causes the next wave of infections and deaths. Putting these factors all together, we can conclude that the performance of these LSTM networks is acceptable in forecasting the emergence of future waves.

## 7 Conclusion

Rapid spread and invasion of COVID-19 worldwide have emphasized the importance of designing models for predicting the epidemiological data for planning strategies to control and prevent the progression of the outbreak. A vast body of research has been devoted to studying the behavior of COVID-19, in terms of the number of infections, deaths, recoveries, hospitalizations, and the basic reproduction number, in different geographical regions considering different time spans and variants of this virus.

In this paper, we have focused on analyzing two noticeably different variants of concern of COVID-19: Alpha and Delta variants. The analysis of these variants are performed considering different geographical regions in terms of size and diversity. We have considered COVID-19 statistics in the country of Iran, the continent of Asia, and the whole world for this analysis. We have examined the predictive power of four different neural network models, namely, ED-LSTM, Bi-LSTM, Conv-LSTM, and GRU to predict the number of infections and deaths for the next three, next five, and next seven days in each of these variants in the aforementioned geographical regions. The results were compared using the evaluation metrics of RMSE, MAE, and MAPE. It is concluded that ED-LSTM and Bi-LSTM are respectively the most powerful models to predict the number of infections and deaths due to Alpha variant of COVID-19. While ED-LSTM still keeps its first rank in predicting the statistics of COVID-19 in Delta variant, GRU takes the second rank in the predictions for Delta variant. Considering different geographical regions, it is concluded that ED-LSTM achieves the best performance in the large geographical regions with great diversity in economical situations and social life style. Putting these factors all together, the Friedman test is used to rank the models. The results show that ED-LSTM, Bi-LSTM, and GRU are in order the best models for predicting the statistics of Alpha and Delta variants of COVID-19, and ED-LSTM is the leading model in all conditions. Hence, we continue examining the power of forecasting of ED-LSTM for the next two months. The results show that ED-LSTM keeps its power in forecasting the future waves. We conclude that ED-LSTM model provides a great performance in predicting the number of infections and deaths due to different variants of COVID-19, and the future similar outbreaks.

In comparison with the earlier results, we should mention a related work of Chandra, Jain, and Singh Chauhan [41] which studied the power of LSTM, Bi-LSTM, and ED-LSTM models in forecasting the number of COVID-19 infections in ten different states of India. They reached the conclusion that although the models showed different performance in the states with different population densities, ED-LSTM generally performed the best for predicting the number of infections in India. They had recommended examining the power of this model in predicting the statistics of COVID-19 in other geographical regions. In this paper, we have shown that ED-LSTM has performed the best for the regions as vast as the continent of Asia and also worldwide. Further, we have shown that besides predicting the number of infections, ED-LSTM also has a great power in predicting the number of deaths due to COVID-19. Moreover, this model keeps its strength in predictions for different COVID-19 variants of concern.

The results of this study can broaden the general knowledge about the behavior of COVID-19 and the power of LSTM networks in predicting the behavior of this virus. These results are useful for epidemiologists, decision makers, healthcare system, and medical device manufacturers to have a clear picture of the future situation of the virus to design efficient health policies to control the virus, and to provide sufficient quantity of the corresponding medical devises and drugs, as well as an adequate number of healthcare providers to treat the patients.

Limitations of the study are as follows. The first and most important limitation on the study is the fact that COVID-19 statistics are only recorded for people with positive PCR tests. However, the reliability of this diagnostic test is under question. The accurate result of this test depends on several factors such as time of the test, the skillfulness of the person who takes samples, and negligence of people who are infected with mild symptoms to take the test. Further, the level of lockdowns, mask-wearing adherence, and social distancing compliance are not considered in this study. Studying these factors as well as considering other effective parameters such as age, gender, and a history of earlier infections are recommended for further research.
